# High HIV prevalence and the internet as a source of HIV-related service information at a community-based organization in Peru: a cross-sectional study of men who have sex with men

**DOI:** 10.1186/s12889-016-3561-4

**Published:** 2016-08-24

**Authors:** R. Colby Passaro, Connie A. Haley, Hugo Sanchez, Sten H. Vermund, Aaron M. Kipp

**Affiliations:** 1 Vanderbilt Institute for Global Health, Vanderbilt University, Nashville, TN USA; 2 Department of Medicine, Vanderbilt University Medical Center, Nashville, TN USA; 3 Epicentro Salud, Lima, Peru; 4 Department of Pediatrics, Vanderbilt University Medical Center, Nashville, TN USA; 5 Institute for Medicine and Public Health, 2525 West End Ave., Suite 614, Nashville, TN USA

**Keywords:** HIV, Testing, Resource-limited, Marginalized populations, Community organizations, Education and outreach, Public health

## Abstract

**Background:**

The HIV prevalence among men who have sex with men (MSM) in Peru (12.4 %) is 30 times higher than in the general adult population (0.4 %). It is critical for community-based organizations to understand how to provide HIV services to MSM while maximizing limited resources. This study describes the HIV prevalence and risk profiles of MSM seeking HIV services at a community-based organization in Lima, Peru. It then compares HIV prevalence between those who found out about the HIV services through different sources.

**Methods:**

A cross-sectional study of MSM seeking HIV services at Epicentro Salud in Lima, Peru for the first time between April 2012 and October 2013. We compared HIV prevalence among MSM who found out about Epicentro via online sources of information (*N* = 419), those using in-person sources (friends, partners) (*N* = 907), and sex workers (*N* = 140) using multivariable logistic regression models.

**Results:**

HIV prevalence was 18.3 % overall: 23.2 % among MSM using online sources, 19.3 % among sex workers, and 15.9 % among MSM using in-person sources. However, when compared to the in-person group, sexual risk behaviors were not statistically higher among MSM using online sources. For the sex worker group, some behaviors were more common, while others were less. After adjusting for confounders, the odds of having HIV was higher for the online group (Odds Ratio = 1.61; 95 % Confidence Interval: 1.19–2.18), but not for the sex worker group (OR = 1.12; 95 % CI: 0.68–1.86), compared to the in-person group.

**Conclusion:**

Internet-based promotion appears to successfully reach MSM at high risk of HIV in Peru. Outreach via this medium can facilitate HIV diagnosis, which is the critical first step in getting infected individuals into HIV care. For community-based organizations working in resource-limited settings, this may be an effective strategy for engaging a subset of high-risk persons in HIV care.

## Background

The estimated prevalence of HIV in Latin America in 2014 was 0.2–0.7 % [1] and is particularly concentrated among urban men who have sex with men (MSM) [2]. According to the 2014 UNAIDS progress report, between 7 and 20 % of MSM in Latin America are infected with HIV [3]. Approximately half of all new infections in the region stem from unprotected anal intercourse (UAI) between men [4, 5]. In 2011, the prevalence of HIV among MSM in Peru was thirty times greater than that in the general adult population (12.4 % vs. 0.4 %) and was most prevalent in the capital city of Lima [2, 6].

Diagnosis is the critical first step in getting infected individuals into HIV care, but less than 50 % of MSM in Lima have been tested for HIV [7]. Among those who do present for testing, as few as 59 % attend a follow-up visit to receive their test results [8]. Moreover, less than a quarter of MSM know the HIV status of at least one of their last three partners [9]. Reported reasons for low testing levels include fear of receiving a positive diagnosis, resulting in lifelong dependency on HIV treatment, drastic lifestyle changes, and even death [7, 10].

The Internet is well established as a mechanism for promoting novel HIV prevention interventions targeting MSM in low and middle-income countries (LMIC) due to its accessibility and cost-effectiveness [11–14]. Peru is classified as an upper-middle income country by the World Bank [15]. According to World Bank data from 2014, 40.2 per 100 people in Peru are internet users, defined as individuals who have used the Internet from any location in the last 12 months via computer, mobile phone, personal digital assistant, games machine, digital TV, etc. [15]. In the last decade, mobile-cellular subscriptions have catapulted from 8.63 to 98 per 100 inhabitants in Peru, per the United Nations International Telecommunication Union [16].

While World Bank data identifies only 40 % of Peruvians as Internet users, studies have reported the utility of the Internet as a tool to access high-risk MSM in Peru as early as 2007 [13, 15]. This study also highlighted that 80 % of these people access the Internet via *cabinas publicas*, or Internet cafes, which are generally concentrated in the same urban areas where most MSM live in Peru [13]. According to a 2011 study, a high-risk subset of men not reached by traditional prevention methods uses the Internet to seek both sexual partners and information about HIV and other sexually transmitted infections (STI) [12].

Because Internet use by MSM is correlated with high-risk sexual behavior and is increasing rapidly in LMICs, understanding factors that distinguish MSM who regularly use online resources related to sexual health from those less likely to use them would enhance strategic planning for public health interventions [13, 17–19]. Community based organizations (CBOs) in resource-limited settings need to know how and where to target their outreach activities to reach the highest risk populations and maximize the impact of limited resources. This study aims to describe both the HIV prevalence and risk profiles of MSM who sought HIV-related servicesat Epicentro Salud (Epicentro), a community-based organization in Lima, Peru, comparing HIV prevalence among those who found out about Epicentro through online media and those who reported sex work to those who became aware of the organization through word of mouth.

## Methods

### Study site

Epicentro is a lesbian, gay, bisexual, and transgender friendly CBO in Lima, Peru offering medical services and STI/HIV testing and counseling in a safe space for approximately 1,000 individuals each year. All persons testing HIV-positive at Epicentro are referred to other local HIV treatment centers such as Impacta, Via Libre, or Cayetano Heredia because Epicentro does not provide HIV treatment and care. However, HIV-positive persons often continue to return to Epicentro for counseling, community events, and other medical care.

### Data collection

All individuals seeking STI/HIV-related services at Epicentro complete a questionnaire that includes basic demographic information, STI/HIV history and prior testing, and sexual risk behavior (at last encounter, within the last 3 months, and within the last 6 months). The questionnaire is two pages, and is administered orally by STI/HIV counselors in Spanish every time the individual comes for testing. All of the questions on the form are closed-ended, except those that require a numerical response.

Questionnaire data from all adults seeking STI/HIV services at Epicentro for the first time between April, 2012 and October, 2013 were used for this cross-sectional study. Biological females and men who reported no history of sex with other men were excluded. Questionnaires were accessed via paper medical records stored at Epicentro and double-entered into Microsoft Excel^™^ after obtaining approval from the agency’s clinic coordinator and executive director. Epicentro routinely uses the Impacta Institutional Review Board in Lima, Peru, for all research projects, thus approval for this study was obtained from the Institutional Review Boards at Impacta and Vanderbilt University in the United States. Written informed consent from participants was not required because the data are collected as part of routine client intake procedures and because all patient identifiers were removed for the analysis.

### Data analysis

Based on an individual’s response to the question “How did you find out about Epicentro services?”, the study population was divided into three groups. MSM who found out about Epicentro from online sources including Facebook, the Epicentro website, or other Internet sites were categorized as the “Online” group, and those who found out about Epicentro from a friend or partner were categorized as the “In-person” group. The third group included participants who indicated their current occupation as sex workers, regardless of how they learned about Epicentro (all but one reported learning about Epicentro from in-person sources). It was felt these individuals would differ substantially from the non-sex workers in terms of risk profile and HIV prevalence, thus warranting a separate category. MSM who reported they learned about Epicentro from educational material (*N* = 70; 5 % of the total sample) were excluded from the final analysis due to the ambiguous nature of this category and the small number of men. We chose to use recruitment to Epicentro by online sources as our exposure variable, rather than Internet use to find sexual partners or health information, because of its implications for informing outreach and education activities by Epicentro.

The primary outcome of interest was HIV prevalence, defined as testing HIV positive on the first visit at Epicentro. Chi-squared tests for categorical variables and non-parametric Mann–Whitney U (Wilcoxon rank-sum) tests for continuous variables were used to compare HIV prevalence, demographic characteristics, and other HIV risk factors among each of the three groups. Logistic regression was used to compare the odds of being HIV-positive for the online and sex worker groups relative to the in-person recruitment group. Variables that were significantly associated with HIV prevalence in bivariate analysis (*p* < 0.05), and other important behavioral factors were included as covariates in the final models.

Two logistic regression models were performed. The first adjusted for age, previous HIV test, sexual role in the past six months, and sexual risk behaviors in the past three months. The second model adjusted for each of the aforementioned covariates plus self-report of the following Internet-based behaviors in the past three months: Internet use to find information about STIs, Internet use to search for sexual partners, and sex with a partner met online. The purpose of the second model was to reveal if any possible differences in HIV prevalence between the in-person and online groups was specifically explained by the reported Internet use behaviors.

## Results

### Descriptive statistics

This study included 907 MSM in the in-person group, 419 in the online group, and 140 in the sex worker group. Participant characteristics are presented in Table 1.

**Table 1:** Demographic, clinical, and behavioral characteristics of MSM seeking HIV testing at a community-based organization in Lima, Peru, 2012–2013

	In person (*N* = 907)	Online (*N* = 419)	Sex Worker (*N* = 140)
HIV-positive, *N* (%)	144 (15.9)	97 (23.2) ^**^	27 (19.3) ^**^
Age, median (IQR)	25 (21–30)	25 (22–30)	24 (20–28) ^**^
Any previous HIV test, *N* (%)	652 (71.9)	316 (75.4)	124 (88.6) ^**^
Sexual role in past 6 months, *N* (%)			
No sex	14 (1.5)	4 (1.0) ^**^	1 (0.7) ^**^
Penetrative	263 (29.0)	93 (22.2) ^**^	6 (4.3) ^**^
Receptive	197 (21.7)	86 (20.5) ^**^	69 (49.3) ^**^
Both receptive and penetrative	433 (47.7)	236 (56.3) ^**^	64 (45.7) ^**^
1 or more STI symptoms in past 6 months^a^, *N* (%)	320 (35.3)	243 (58.0) ^**^	37 (26.4) ^**^
Number of sex partners in past 3 months, median (IQR)	3 (1–5)	2 (1–4) ^**^	110 (30–250) ^**^
Gender of sex partners, *N* (%)			
No partners in last 3 months	69 (7.6)	48 (11.5) ^**^	0 (0) ^**^
Men only	673 (74.2)	337 (80.4) ^**^	133 (95) ^**^
Men and women	132 (14.6)	14 (3.3) ^**^	6 (4.3) ^**^
Women only	13 (1.4)	5 (1.2) ^**^	0 (0) ^**^
Sex partner had STI, *N* (%)	101 (11.1)	40 (9.55)	38 (27.1) ^**^
Consistency of condom use in past 3 months^b^, *N* (%)			
100 % condom use	273 (30.1)	138 (32.9)	34 (24.3) ^**^
50–99 % condom use	230 (25.4)	84 (20.0)	94 (67.0) ^**^
1–49 % condom use	218 (24.0)	106 (25.3)	10 (7.1) ^**^
Never used condom	186 (20.5)	91 (21.7)	2 (1.4) ^**^
Alcohol/drug use during sex in past 3 months, *N* (%)	343 (37.8)	121 (28.9) ^**^	70 (50.0) ^**^
Exchanged money or goods for sex in past 3 months, *N* (%)	172 (19.0)	16 (3.82) ^**^	138 (98.6) ^**^
Looked up STI information online in past 3 months, *N* (%)	310 (34.2)	219 (52.3) ^**^	15 (10.7) ^**^
Used the Internet to look for sex in past 3 months, *N* (%)	247 (27.2)	160 (38.2) ^**^	35 (25.0)
Sex with partner met on the Internet in past 3 months, *N* (%)	263 (29.0)	186 (44.4) ^**^	35 (25.0)

** *p* < 0.05 when compared to in-person group; chi-square for categorical variables; non-parametric Mann–Whitney *U* test for age and number of sexual partners

^a^At least one of the following: Burning with urination, warts, secretion, or ulcers

^b^Calculated as the number of sexual encounters in which a condom was not used, divided by the total number sexual encounters

#### HIV prevalence and testing history

The overall HIV prevalence for MSM among this study population was 18.3 %, but varied by sub-group: 15.9 % for MSM who found out about Epicentro by word of mouth, 23.2 % for MSM who learned about Epicentro online, and 19.3 % for sex workers. A majority of the men and transgender women seeking HIV-related services at Epicentro also reported previous HIV testing: 71.9 % of the in-person group, 75.4 % of the online group, and 88.6 % of the sex workers.

#### Sexual role

Most participants in all three groups reported having sex in the past six months (>98.0 % in each group). Overall, a versatile sexual role of both penetrative and receptive intercourse was most commonly reported (50 %). The distribution of sexual roles was similar for the in-person and online groups: penetrative (29.0 % vs. 22.2 %), receptive (21.7 % vs. 20.5 %), and both (47.7 % vs. 56.3 %). In contrast, the majority of sex workers reported either receptive (49.3 %) or versatile roles (45.7 %).

#### Sexual risk behavior in the last three months

The proportion of participants with one or more STI symptoms in the last six months was substantially higher in the online group (58 %) compare to either the in-person group (35 %) or the sex worker group (26 %). The median number of sex partners in the last three months was low in both the in-person and online groups (3 vs. 2 partners) but much higher among the sex workers (110 partners). Notably, the proportion of sex workers reporting condom use in more than half of their sexual encounters in the last three months was much greater compared to the other groups (91 % versus 56 % and 53 % in the in-person and online groups, respectively). Only 1.4 % of sex workers reported never using a condom in their penetrative sexual encounters in the last three months, while 20.5 % of the in-person group and 21.7 % of the online group reported not using condoms at all during this same period.

With regards to the question of whether money or goods were exchanged for sex in the previous three months, nearly 100 % of the sex worker group affirmed this behavior. We therefore excluded this risk factor from the logistic regression modeling to avoid unstable estimates. Interestingly, the in-person group reported a much higher frequency of exchange of money or goods for sex in the preceding three months (19.0 %) compared to the online group (3.8 %). In regards to both alcohol/drug use and sex with a partner with an STI, sex workers reported the highest frequencies (50 % and 27 %, respectively), compared to approximately one-third reporting alcohol and/or drug use and 10 % reporting sex with a partner with an STI in each of the other groups.

#### Internet use in the last three months

The online group had the highest positive response rate for all three measures related to the Internet in the last three months: sex with an Internet partner (44.4 %), using the Internet to look up STI information (52.3 %), and using the Internet to look for sex (38.2 %). In this context, an “Internet partner” was defined as any partner the subject met on the Internet. Reported frequencies for all three of these measures were less than 35 % in the other two groups (Table 1).

### Statistical modeling

Table 2 shows results from the crude and two adjusted logistic regression models. The crude odds of being HIV positive among the online group was 1.60 times that of the in-person group (95 % CI, 1.20–2.13) and did not change substantially after adjusting for the initial set of covariates (OR = 1.58; 95 % CI: 1.18–2.13) or further adjustment for Internet use (OR = 1.61; 95 % CI: 1.19–2.18). Other risk factors significantly associated with testing HIV positive include not having a prior HIV test and engaging in receptive anal intercourse (versus penetrative) or both receptive and penetrative anal intercourse. All other risk factors showed minimal or no association with HIV prevalence.

**Table 2:** Crude and adjusted logistic regression models for the association between recruitment type and testing HIV positive at a community-based organization in Lima, Peru, 2012–2013

Characteristic/Behavior	Crude		Adjusted			
	OR^a^	(95 % CI)	OR	(95 % CI)	OR	(95 % CI)
In person	Ref		Ref		Ref	
Online	1.60	(1.20, 2.13)	1.58	(1.18, 2.13)	1.61	(1.19, 2.18)
Sex Worker	1.27	(0.80, 2.00)	1.12	(0.68, 1.84)	1.12	(0.68, 1.86)
Age	1.01	(0.99, 1.02)	1.01	(0.99, 1.03)	1.01	(0.99, 1.03)
Any previous HIV test^b^	0.70	(0.52, 0.93)	0.62	(0.45, 0.84)	0.62	(0.45, 0.85)
Sexual Role in past 6 months						
Penetrative	Ref		Ref		Ref	
Both receptive and penetrative	2.13	(1.44, 3.14)	2.08	(1.40, 3.08)	2.09	(1.40, 3.10)
Receptive	1.78	(1.14, 2.78)	1.67	(1.06, 2.63)	1.66	(1.05, 2.62)
No sex	1.42	(0.46, 4.33)	1.31	(0.43, 4.04)	1.34	(0.43, 4.14)
Consistency of condom use in past 3 months^c^						
100 % condom use	Ref		Ref		Ref	
50–99 % condom use	1.09	(0.77, 1.55)	1.09	(0.75, 1.58)	1.09	(0.75, 1.59)
1–49 % condom use	1.07	(0.74, 1.55)	1.00	(0.68, 1.46)	0.96	(0.66, 1.41)
Never used condom	1.05	(0.71, 1.55)	0.97	(0.65, 1.46)	0.95	(0.63, 1.43)
Alcohol/drug use during sex in past 3 months	1.13	(0.86, 1.49)	1.16	(0.87, 1.55)	1.18	(0.89, 1.57)
Sex partner had STI in past 3 months	1.24	(0.84, 1.82)	1.27	(0.85, 1.91)	1.25	(0.83, 1.88)
Looked up STI information online in past 3 months	0.97	(0.73, 1.29)			0.68	(0.42, 1.08)
Used the internet to look for sex in past 3 months	1.16	(0.88, 1.51)			1.09	(0.80, 1.50)
Sex with partner met on the internet in past 3 months	1.09	(0.82, 1.46)			1.28	(0.80, 2.06)

^a^The reported crude odds ratios (ORs) represent a series of individual logistic regressions depicting the relationships between the risk factors and the outcome variable (HIV status), i.e., models with only one risk factor being analyzed

^b^Responding “No” to the dichotomous variables is the referent group

^c^Calculated as the number of sexual encounters in which a condom was not used, divided by the total number sexual encounters

## Discussion

We found that nearly 23 % of Peruvian MSM who found out about HIV services at Epicentro via online sources were HIV-infected. This was substantially higher than other MSM who received the same information via friends or partners (16 %), a significant difference even after accounting for confounding factors including Internet behaviors (aOR = 1.61; 95 % CI: 1.19–2.18). This population of MSM represents an important group for HIV surveillance and prevention activities. As expected, the observed HIV prevalence in the sex worker group was also higher (19 %), but did not differ substantially from MSM who received the same information via friends or partners.

The overall HIV prevalence of 18.3 % from this study falls within the range previously reported by studies of MSM in Lima (10–30 %) for which participant recruitment varied across studies (e.g., general adult clinics, clinics catering more to MSM, or community-based recruitment) [20–23]. Of additional interest, most sexual risk profiles between the in-person and online groups were similar, but the online group was less likely to use alcohol and drugs during sex or to exchange money or goods for sex, while more often reporting internet-related behaviors, as expected. These differences, however, did not explain the observed association between finding out about Epicentro via online sources and having a higher HIV prevalence. Thus, MSM who were responsive to online information about Epicentro services may represent a much broader, but still at-risk, group of MSM than those who specifically seek out sexual partners online.

Among MSM who identified as sex workers, the observed HIV prevalence (19.3 %) falls between that of the in-person (15.9 %) and online (23.2 %) groups. Not surprisingly, sex workers reported more frequent sexual risk behaviors, yet they also reported higher use of HIV prevention behaviors, including HIV testing and regular condom use. This group also reported lower prevalence of STI symptoms in the last 6 months. These findings are consistent with recent studies looking at condom use and other prevention strategies employed by MSM sex workers and their clients in Peru and other parts of the developing world [24–26]. The high frequency of risk prevention techniques reported by sex workers is a clear sign that STI prevention outreach and education to this high-risk group has had an impact, and the lessons learned from this work could potentially be applied to other groups.

Findings from this study specifically suggest that MSM who actively engage online media (for example, to identify MSM or HIV-related services), even for reasons other than finding sexual partners, are at increased risk of HIV infection. Numerous studies looking at the risk profiles of MSM in the United States and other high income countries suggest those who use the Internet to look for sex have an increased risk for HIV infection due to the anonymity provided by this media. However, few studies have sought to corroborate these results in a low-resource setting [17–19, 27–30]. The reason why our study found engaging online media for HIV-service information to be related to risk of testing positive for HIV, independent of Internet use to find sexual partners, is not fully clear. It may reflect concerns about remaining anonymous about one’s sexual orientation and HIV risk, which in turn may affect HIV service utilization, rather than specific behaviors that increase risks of HIV infection directly. Some studies have suggested that stigma, discrimination, or disclosure concerns around sexual orientation may negatively impact HIV prevention and services utilization [31–33].

Our finding that MSM who reported using online media as one of their sources of HIV-related information have a substantially higher prevalence of HIV has important implications for how public, private and not-for-profit agencies strategically plan their HIV prevention and testing service delivery and marketing. Targeting resources via online and social media routes facilitate access to an important risk group with increased need of HIV testing and subsequent treatment or prevention interventions. A small but growing literature has begun to explore the use of web-based HIV interventions via randomized controlled trials (RCT) or quasi-experimental studies among MSM and conducted entirely over the Internet [11]. Most studies are from the United States or other high-income countries and report a reduction in one or more risk behaviors or increases in HIV testing, knowledge, self-efficacy, outcome expectancies, and/or disclosure [11, 34, 35]. One small RCT (*n* = 187), conducted in Peru, found a 5-min online video successfully moved participants through stages of change for HIV testing (e.g., contemplation to preparation stage) and was associated with both making a testing appointment and receiving an HIV test among non-gay identified MSM (no association among gay-identified MSM) [14]. The advantages of online interventions lie in their ability to engage large numbers of individuals from hard-to-reach populations such as MSM in a consistent and cost-effective manner, while providing the anonymity that is not possible with clinic- or peer-based interventions [11]. The combined effects of increased uptake of modern technology and personalized developments in HIV prevention strategy, including things like the release of the OraQuick^TM^ in-home HIV test in the United States, should make it easier for MSM reluctant to access services in-person to take advantage of similar services in the privacy of their own homes.

This study has some limitations. First, the study used a cross-sectional design, which limits inference about a causal relationship. Still, the high HIV prevalence among the online group is concerning and indicates the need for ongoing prevention and testing messages geared towards MSM using online social media and other sites. Second, the study data were collected for routine clinical and testing purposes, and consequently did not have sufficient detail to more fully explore Internet use for HIV information or risky sexual behaviors. Third, the Epicentro questionnaire asked for information regarding all sexual risk behaviors and reported Internet use in the *past three months*, whereas questions referring to sexual role pertained to the *past six months*. It is unlikely, however, that the difference in these time periods would add any bias to study results. Finally, there is a possibility for misrepresentation inevitable in any study relying on self-report, whether this is due to shame, recall bias, intent to please the counselor conducting the survey, or just from providing quick responses without careful consideration. For example, it is possible that there are some sex workers in the in-person group that did not identify themselves as such. Misclassified sex workers would likely be in the in-person group (reporting having heard about Epicentro through a friend) because all of the sex workers except one originally fell into that group prior to being separated as an individual category. Such misclassification would likely inflate the HIV prevalence of the in-person group, meaning that the observed association between finding out about Epicentro HIV-related services via the Internet and HIV prevalence may be underestimated.

## Conclusion

Increasing Internet uptake in low-resource settings where there is high HIV prevalence among MSM represents a unique opportunity for CBOs in this setting to employ low-cost, internet-based HIV prevention and testing messages. In the context of Lima, Peru, education and outreach for HIV/STI prevention and testing has traditionally been conducted through peer education, which requires investment of substantial human and financial resources. The transition of some of this messaging to Internet marketing may permit these CBOs and other public health entities to focus limited resources as well as broaden the number of individuals reached, particularly among high-risk groups such as MSM who would benefit the most. Further research is needed to explore the differential risk profiles for MSM that actively use the Internet as a resource for information about sexual health and to seek sexual encounters so that interventions can be more specifically tailored to the target population. Organizations providing HIV prevention and testing services should also continue to test Internet-based marketing to evaluate both its reception and usability in this unique MSM population.
